# NR4A3, a possibile oncogenic factor for neuroblastoma associated with CpGi methylation within the third exon

**DOI:** 10.3892/ijo.2014.2340

**Published:** 2014-03-13

**Authors:** SHOTA UEKUSA, HIROYUKI KAWASHIMA, KIMINOBU SUGITO, SHINSUKE YOSHIZAWA, YUI SHINOJIMA, JUN IGARASHI, SRIMOYEE GHOSH, XAOFEI WANG, KYOKO FUJIWARA, TARO IKEDA, TSUGUMICHI KOSHINAGA, MASAYOSHI SOMA, HIROKI NAGASE

**Affiliations:** 1 Departments of Pediatric Surgery and, Nihon University, Tokyo, Japan;; 2 Cancer Genetics, School of Medicine, Nihon University, Tokyo, Japan;; 3 Life Science Advanced Research Institute for the Sciences and Humanities, Nihon University, Tokyo, Japan;; 4 Department of Zoology, North-Eastern Hill University, Meghalaya, India;; 5 Chiba Cancer Center Research Institute, Chiba;; 6 Innovative Therapy Research Group, Nihon University Research Institute of Medical Science; 7 Division of General Medicine, Department of Medicine, Nihon University School of Medicine, Tokyo, Japan

**Keywords:** nuclear receptor subfamily 4, group A, member 3, DNA methylation, neuroblastoma

## Abstract

Aberrant methylation of *Nr4a3* exon 3 CpG island (CpGi) was initially identified during multistep mouse skin carcinogenesis. *Nr4a3* is also known as a critical gene for neuronal development. Thus, we examined the *Nr4a3* exon 3 CpGi methylation in mouse brain tissues from 15-day embryos, newborns and 12-week-old adults and found significant increase of its methylation and Nr4a3 expression during mouse brain development after birth. In addition, homologous region in human genome was frequently and aberrantly methylated in neuroblastoma specimens. A quantitative analysis of DNA methylation revealed that hypomethylation of CpG islands on *NR4A3* exon 3, but not on exon 1 was identified in three neuroblastomas compared with matched adrenal glands. Additional analysis for 20 neuroblastoma patients was performed and 8 of 20 showed hypomethylation of the CpGi on *NR4A3* exon 3. The survival rate of those 8 patients was significantly lower compared with those in patients with hypermethylation. Immunohistochemical NR4A3 expression was generally faint in neuroblastoma tissues compared with normal tissues. Moreover, the MYCN amplified NB9 cell line showed hypomethylation and low expression of *NR4A3*, while the non-MYCN amplified NB69 cell line showed hypermethylation and high expression. These results indicate that DNA hypomethylation of the CpGi at *NR4A3* exon 3 is associated with low *NR4A3* expression, and correlates with poor prognosis of neuroblastoma. Since NR4A3 upregulation associated with the hypermethylation and neuronal differentiation in mice, poor prognosis of neuroblastoma associated with NR4A3 low expression may be partly explained by dysregulation of its differentiation.

## Introduction

Neuroblastoma is an embryonic tumor of neuroectodermal cells derived from the neural crest. It is the most common extracranial solid tumor in children, and it accounts for approximately 15% of all pediatric oncology death. Survival rate of the patient with high-risk neuroblastoma is still <40%, despite combined modality therapy (1,2).

Recent advances have disclosed the significance of epigenetic events in the development and progression of human tumorigenesis. Generally, global DNA methylation levels are low in cancer and has been linked to genomic instability, which can lead to DNA damage. On the other hand, promoter-specific hypermethylation of specific genes, such as tumor suppressor genes, is the most common event in tumorigenesis (3). It is also reported that predetermined epigenetic program provides required direction for the number of changes during embryonic and postnatal development that are necessary for proceeding from an oocyte to a fully developed adult animal (4). DNA methylation is the one of the best-characterized epigenetic modifications and plays an important role in the diverse genomic processes, such as gene regulation, chromosomal stability, parental imprinting and X-inactivation (5). Recent genome-wide DNA methylation searches indicate that 4 to 17% of CpG sites are different in methylation among tissues and developmental processes (6–8). The methylation status at the tissue-specific differentially methylated regions (T-DMRs) and developmental-specific differentially methylated regions (DS-DMRs) are suggested to play important roles in development and differentiation.

Nuclear receptor subfamily 4, group A, member 3 (*Nr4a3*), also known as neuron-derived orphan receptor 1 (NOR1), is a member of NR4A subgroup of orphan nuclear receptor. In mammals, the NR4A subgroup consists of NR4A1 (Nur77), NR4A2 (Nurr1) and NR4A3 (9). The monomer form of those receptors binds to the nerve growth factor-induced clone B response element (NBRE), and homodimer or heterodimer forms bind to the Nur1 response element in the promoter of their target genes which may be essential for the development of dopaminergic neurons in the midbrain (10). We identified the CpG sites in *Nr4a3* exon 3 as a mouse skin cancer T-DMR and a mouse brain DS-DMR by using analyses of restriction landmark genomic scanning (RLGS) and methyl-DNA immunoprecipitation (MeDIP), respectively (Fujiwara *et al*, unpublished data). Here, we analyzed involvement of DNA methylation at *Nr4a3* exon 3 CpGi in Nr4a3 expression, mouse brain development, neuroblastomagenesis and association with its poor prognosis.

## Materials and methods

### Tissue samples

C57 BL/6J mice were purchased from Jackson Laboratory (Bar Harbor, ME) and maintained in Oriental Yeast Co. Ltd (Tokyo, Japan). Brain specimens from mice at three different developmental stages: E15, 15-day-old embryo; NB, new born; and AD, 12-week adult; were disected and stored as described previously (11).

Twenty primary neuroblastoma tumors were obtained in Nihon University Hospital (Tokyo, Japan) at the time of diagnosis, from 1999 to 2007. All the analyses of those specimens were performed under the approval of Nihon University Institutional Review Boards (IRB no. 51). Neither neoadjuvant chemotherapy nor irradiation therapy was given preoperatively to any patient. Four adrenal samples were collected from a nephroblastoma patient undergoing nephrectomy and from 3 neuroblastoma patients (cases 3, 8 and 20) undergoing tumor resection. All of the samples were immediately snap-frozen in liquid nitrogen and stored at −80°C until use. Summary of these patients is shown in Table I.

### Cell lines and culture condition

Human neuroblastoma cell lines NB9 and NB69 were obtained from Riken Cell Bank (Tsukuba, Japan). Both human neuroblastoma cell lines were maintained in RPMI-1640 (Nalarai Tesque, Kyoto, Japan) supplemented with 15% fetal bovine serum (Nichirei Biosciences, Tokyo, Japan), 100 IU/ml penicillin (Gibco™, Carlsbad, CA) and 100 *μ*l/ml streptomycin (Gibco). The cells were cultured in a 37°C humidified atmosphere containing 5% CO_2_ maintained in appropriate conditions recommended by the manufacturers.

### DNA preparation and bisulfite treatment

Total genomic DNA was extracted from mouse brains, primary tumors, neuroblastoma cell lines and normal adrenal medullas with DNeasy Tissue Kit (Qiagen, Valencia, CA) and modified by sodium bisulfite using the EZ DNA Methylation Kit (Zymo Research, Orange, CA), by following the manufacturer’s instructions.

### Quantitative analysis of DNA methylation using base-specific cleavage and matrix-assisted laser desorption/ionization time-of-flight mass spectrometry (MALDI-TOF MS)

Sequenom MassARRAY quantitative methylation analysis (12) using the MassARRAY Compact System (www.sequenom.com) was employed for the quantitative DNA methylation analysis at CpG dinucleotides. This system utilizes mass spectrometry (MS) for the detection and quantitative analysis of DNA methylation using homogeneous MassCLEAVE (hMC) base-specific cleavage and matrix-assisted laser desor ption/ionization time-of-f light (MALDI-TOF) MS (13). The MethPrimer program (http://www.urogene.org/methprimer/index1.html) (12) was used to design bisulfite PCR primers (Table II). Each reverse primer has a T7-promotor tag for *in vitro* transcription (5′-cagtaatacgactcactatagggagaaggct-3′), and the forward primer is tagged with a 10 mer to balance melting temperature (TM) (5′-aggaagagag-3′). All primers were purchased from Operon (Tokyo, Japan). Polymerase chain reaction (PCR) amplification was performed using HotStarTaq Polymerase (Qiagen) in a 5 *μ*l reaction volume using PCR primers at a 200 nM final concentration, and bisulfate treated DNA (∼20 ng/ml). After the treatment of shrimp alkaline phosphatase, 2 *μ*l of the PCR products was used as a template for *in vitro* transcription and RNase A Cleavage for the T-reverse reaction (3′ to either rUTP or rCTP), as described in the manufacturer’s instructions (Sequenom hMC, Sequenom, San Diego, CA). The samples were desalted and spotted on a 384-pad SpectroCHIP (Sequenom) using a MassARRAY nanodispenser (Samsung Seoul, Korea), followed by spectral acquisition on a MassARRAY Analyzer Compact MALDITOF MS (Sequenom). The resultant methylation calls were analyzed by EpiTYPER software v1.0 (Sequenom) to generate quantitative measurements for each CpG site or an aggregate of multiple CpG sites. Since maldi-TOF mass methylated peaks do not denote a particular CpG site, but rather corresponds to the number of CpG sites methylated within the cleavage fragment, we decided to present average percent methylation of all CpG sites in the bisulfite PCR fragment with the standard curve.

Standard curve of DNA methylation level was made by using 0, 25, 50, 75 and 100% methylated samples. BAC DNA (RPMI-11 341L6) obtained from Roswell Park Cancer Institute (Buffalo, NY) was used as 0% methylation and M.Sss-1 double treated BAC DNA was used as 100% methylation. The PCR was carried out with a final volume of 50 *μ*l, containing 1.0 *μ*l of each 10.0 *μ*M primer (final concentration 0.2 *μ*M), 8.0 *μ*l of 2.5 mM dNTP, 25 *μ*l of 2 times GC Buffer (Takara Bio, Shiga, Japan), 0.5 U of LA taq (Takara Bio) and 1 *μ*l of genomic DNA as a template. Amplification was carried out with an initial denaturing at 94°C for 1 min followed by 45 cycles of denaturing at 94°C for 30 sec, annealing for 1 min at the annealing temperature of each primer (60°C), extension for 3 min at 72°C, and then a final extension for 5 min at 72°C. The methylation reactions were carried out in 1X M.SssI buffer with 160 *μ*M SAM (New England Biolabs, Ipswich, MA). In total reaction volume of 50 *μ*l, 500 ng PCR product was treated with 4U M.SssI for 1 h at 37°C. Reactions were stopped for 20 min at 65°C and PCR product was purified Qiagen PCR purification kit (Qiagen). This CpG methyltransferase reaction was performed twice. Then M.SssI treated PCR product was produced (14). Curve was fitted and methylation levels were modified.

### Western blot analysis

All of samples were collected and total cell lysates were prepared in M-PER mammalian protein extraction reagent (Thermo, Rockford, IL) containing a protease-inhibitor cocktail (Nalarai Tesque). Proteins (20 *μ*g) were loaded on NuPAGE + 10% Bis-Tris gels (Invitrogen Life Technologies, Carlsbad, CA) for electrophoresis. The proteins were separated at 100 mA for 1 h, then transferred to polyvinylidene difluoride membranes by using iblot transfer for 7 min (Invitrogen). The membranes were incubated with Tris-buffered saline (TBS), containing 5% non-fat milk, 0.2% Tween-20 and a rabbit anti-NR4A3 polyclonal antibody (1:100) overnight (SC-30154, Santa Cruz Biotechnology, Santa Cruz, CA). The membranes were washed three times with a TBS containing 0.2% Tween-20. The immunocomplexed proteins were identified by reaction with a peroxidase-linked goat antibody to rabbit IgG (GE Healthcare, Little Chalfont, UK). Then these immunocomplexed proteins were detected by enhanced chemiluminescent reaction (Amersham Bioscience Inc., Piscataway, NJ). Immunoblotting with antibody to actin (Abcam, Cambridge, MA) provided an internal control for equal protein loading. Chemiluminescent detection was performed by LAS4000 (Fujifilm, Tokyo, Japan).

### Immunohistochemical staining

Formalin-fixed, paraffin-embedded serial sections (4 *μ*m) were deparaffinized in xylene, rehydrated through graded alcohols, and immersed for 15 min in phosphate-buffered saline (PBS). The sections were soaked in 10 mmol/l of sodium citrate buffer (pH 6.9) and treated in a microwave for 15 min for antigen retrieval. After antigen retrieval the endogenous peroxidase activity was blocked with 3% hydrogen peroxidase in methanol for 30 min, and non-specific staining was then blocked by 1-h incubation with normal goat serum (Nichirei Biosciences). The sections were then incubated overnight at 4°C with 2 *μ*g/ml of a rabbit anti-NR4A3 polyclonal antibody (SC-30154: Santa Cruz Biotechnology). The sections were treated for 30 min at room temperature with goat secondary antibody against rabbit immunoglobulins (Nichirei Biosciences). The sections were stained at room temperature for 25 min with AEC substrate kit (Vector Laboratories, Burlingame, CA). After staining with AEC substrate kit the sections were counterstained with hematoxylin.

### Chromatin immunoprecipitation assays

Chromatin immunoprecipitation assays were performed essentially as previously described (15–17) with the following minor modifications. Neuroblastoma cell lines (NB9 and NB69) were fixed in 0.33 M (1%) formaldehyde for 10 min, before adding 4 volumes of ice-cold PBS containing 0.125 M glycine, to give an approximately 2-fold molar excess of glycine over formaldehyde. Then cells were washed with cold PBS containing Protease K (Nalarai Tesque). Crude cell lysates were sonicated to generate 200–1,000 bp DNA fragments. Chromatin was immunoprecipitated with 10 *μ*l of rabbit antiserum raised against human CTCF (07–729; Millipore, Billerica, MA) per 1×10^7^ cells or with 2 *μ*g normal rabbit immunoglobulin G (IgG; Millipore) used as a control, according to manufacturer’s protocols (Millipore). PCR amplification was performed using AccuPrime (Invitrogen) in a 25 *μ*l reaction volume using PCR primers at 200 nM final concentration and 5 *μ*l immunoprecipitated DNA as a template. Amplification was carried out with an initial denaturing at 94°C for 1 min followed by 38 cycles of denaturing at 94°C for 30 sec, annealing for 30 sec at the annealing temperature of each primer, extension for 1 min at 68°C, and then a final extension for 5 min at 72°C, using specific primers as follows: for *NR4A3* exon 3, 5′-CTTCCCGCTCTTCCACTTC-3′; and 5′-TCACCTTGAAAAAGCCCTTG-3′, Tm 58°C; for cMYC, 5′-GTTTTAAGGAACCGCCTGTCCTTC-3′ and 5′-GGA TTGCAAATTACTCCTGCCTCC-3′, Tm 62°C (18). All primers were purchased from Operon.

### Statistical analysis

The Mann-Whitney U test was used to evaluate the statistical significance of the difference in the methylation level of *NR4A3* among the samples. The methylation levels were categorized by Youden index using 17 patients passed the observation period (19). The cutoff point between high and low levels of DNA methylation at each DMR was calculated by ROC curve analysis. Survival curves were calculated according to Kaplan-Meier analysis and compared with a log-rank test. Event-free survival was calculated as the time from diagnosis to event or last examination if the patient had no event. Recurrence, progression of disease and death from disease were counted as events. Death resulting from therapy complications or from second malignancy was not counted as an event but censored for event-free survival. The data were analyzed by the SPSS (Chicago, IL) for Windows. Differences were considered significant at p<0.05.

## Results

### Methylation levels at CpG sites of Nr4a3 exon 3 CpGi and its expression in mouse brain specimens

**Figure 1.**
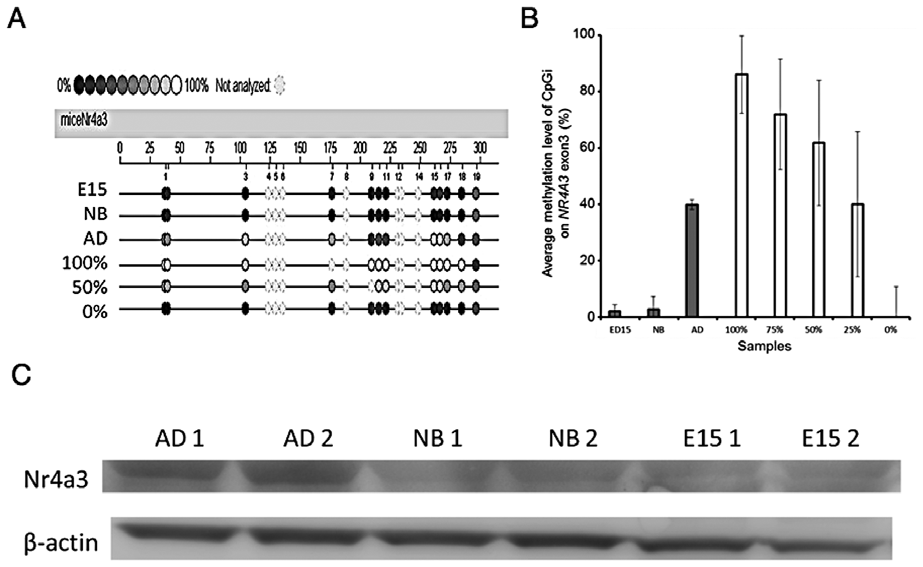
DNA methylation and protein expression levels of NR4A3 in mouse brain samples. DNA methylation level was analyzed quantitatively using Sequenom MassARRAY EpiTYPER. Methylation level is shown in Epigram (A) and average methylation levels are shown in the bar graph (B). Gray columns indicate mouse brain samples and open columns are the standard. Error bars indicate SD. Methylation levels in AD mouse brain samples were significantly higher than those in NB and E15 brains. (p<0.030). (C) NR4A3 and loading control of β-actin protein expression was analyzed by western blotting. AD brain showed higher expression level of NR4A3 than in NB and E15.

Methylation levels of each CpG site at the *Nr4a3* exon 3 CpG island in mouse brains were analyzed at three different developmental stages. This CpGi (mouse chr4:48072571-48072905 in the USCS database, February, 2006 assembly) showed higher methylation level in the brain specimens of 12-weeks-old mice (AD brain), compared with brain specimens from 15-days-old embryos (E15) or new-born mice (NB) in the analysis using Mass ARRAY EpiTYPER (Fig. 1A). The average methylation level of all CpG sites in this region was significantly higher in AD brain than in E15 and NB brain specimens (Fig. 1B). Western blot analysis revealed higher expression level of NR4A3 protein in AD brain specimens, compared with the other developmental stages (Fig. 1C).

### Search for the most different somatic change within homologous Nr4a3 exon 3 CpGi in human neuroblastoma

**Figure 2.**
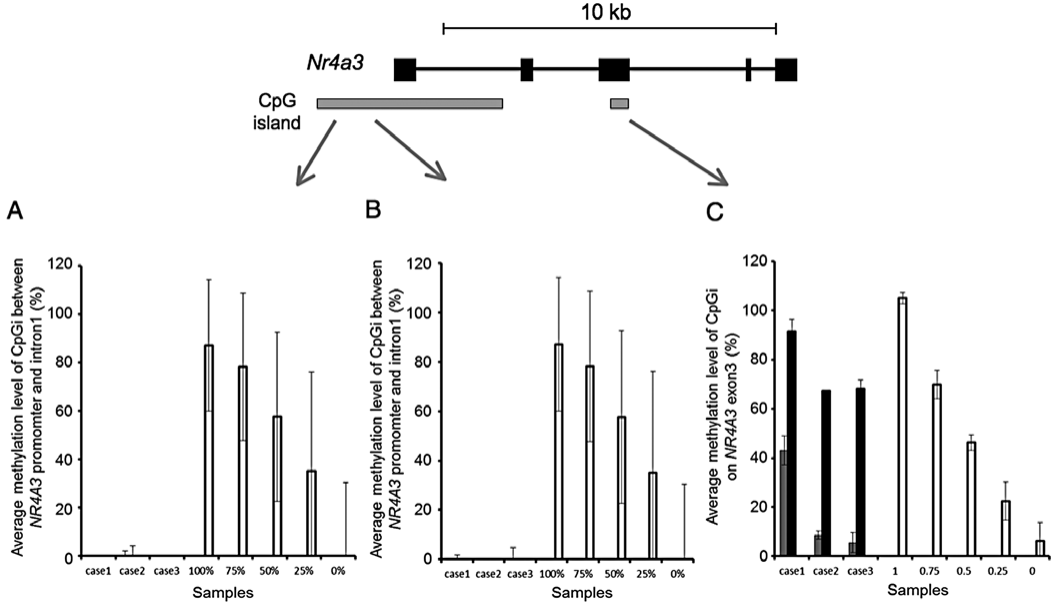
DNA methylation levels of the *NR4A3* region in human neuroblastoma specimens. Methylation levels of human homologous region of *Nr4a3* were analyzed quantitatively using Sequenom MassARRAY EpiTYPER in human neuroblastoma. Methylation levels of CpGi at NR4A3 exon 3 (C), but not in 5′ promoter CpGi (A and B), were significantly higher in neuroblastoma than those in corresponding adrenal glands (p<0.01). Gray columns indicate neuroblastoma samples, black columns are for adrenal samples and open columns are the standard. Data are shown as mean ± SD.

We analyzed methylation level of human homologous region (chr4:48072371-48072905, in USCS genome database, March, 2006, NCBI36/hg18) in the surgical resected NB specimens and found that all 3 NB specimens showed significantly lower methylation level at *NR4A3* exon 3 CpGi compare to those from the matched adrenal tissues (Fig. 2). The CpGi located between *NR4A3* promoter and intron 1 was not methylated at all in either neuroblastoma or adrenal samples.

### Aberrant methylation at NR4A3 exon 3 CpGi confirmed by using Mass ARRAY EpiTYPER method in additional 17 neuroblastoma specimens

**Figure 3.**
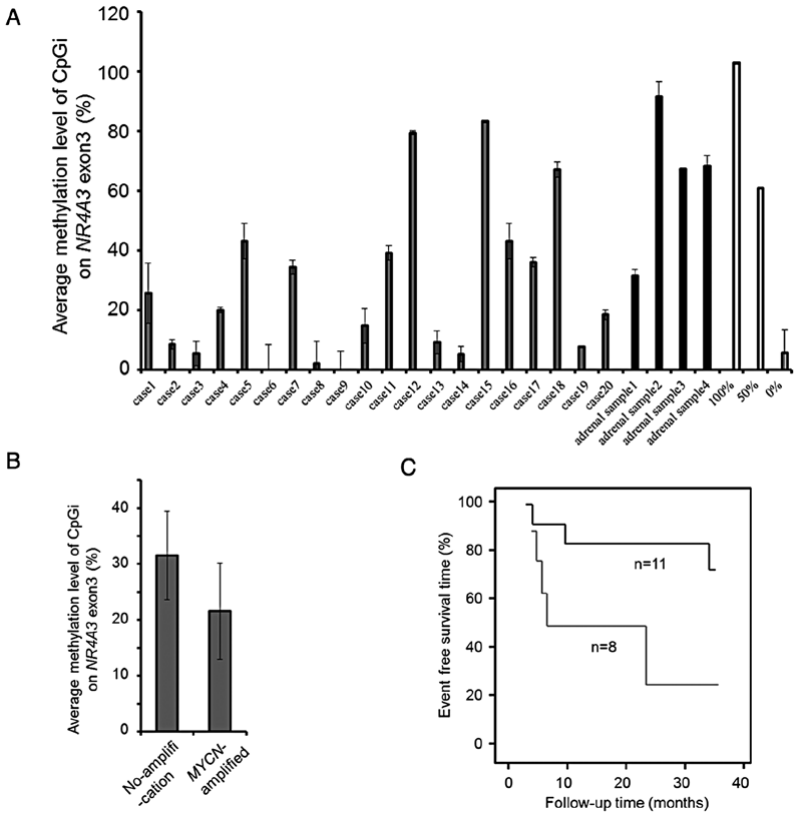
DNA methylation levels of *NR4A3* exon 3 CpGi in additional human neuroblastoma specimens and survival analyses of neuroblastoma patients. Methylation levels in *NR4A3* exon 3 CpGi were analyzed by using Mass ARRAY EpiTYPER method in 20 neuroblastoma specimens and 4 adrenal samples. (A) The bar graphs show the average of methylation levels in the region. Gray columns indicate neuroblastoma samples, black columns are for adrenal samples and open columns are the standard. The error bars indicate SD. (B) Average methylation level of CpGi at NR4A3 exon 3 in 11 samples without MYCN amplification and 9 samples with MCYN amplification are shown. The error bar indicate SEM. (C) Kaplan-Meier analysis was performed to see whether methylation level at NR4A3 exon 3 CpGi associate with the survival length of neuroblastoma patients. Twenty neuroblastoma specimens were segregated into two groups depending on their methylation levels of the *NR4A3* exon 3 regions (hypermethylation, methylation level is higher than 11.93%; hypomethylation, methylation level is 11.93% or lower). Eight out of 20 neuroblastoma specimens were in the hypomethylation tumor group. Black line indicates the hypermethylation group and gray line is for the hypomethylation group. There was a significant association between methylation levels and patient outcome (p=0.034, log-rank test).

Additional 17 neuroblastoma samples and 1 adrenal sample were used to confirm aberrant methylation at *NR4A3* exon 3 CpGi. Adding a new adrenal sample, all the 4 adrenal samples were hypermethylated at *NR4A3* exon 3 CpGi. The average methylation level in 4 adrenal samples was 64.6±2.1%. The methylation level of neuroblastoma specimens varied from *83.3±.. to *−0.5±0.9%, and the average value of the all samples was 27.0±25.8%, which was significantly lower than the average methylation level of 4 normal samples (p=0.005, Mann-Whitney U test) (Table I, Fig. 3A). In 17 out of 20 neuroblastoma specimens, methylation levels at *NR4A3* exon 3 CpGi were low, compared with the average methylation level in 4 adrenal samples. Methylation level in 9 *MYCN* amplified neuroblastoma specimens was significantly lower, compared with that in 11 specimens without *MYCN* amplification (Fig. 3B) (p=0.005, Mann-Whitney U test).

For Kaplan-Meier analysis, cut off value of the methylation level was calculated as 11.93% by using youden index and the methylation levels of 20 patients passed the observation period. Twenty neuroblastoma patients were divided into two groups depending on their methylation levels at the *NR4A3* exon 3 regions. The hypermethylation group has methylation level higher than the cut off value, and the hypomethylation group showed lower than that. Eight out of 20 neuroblastoma specimens were classified to hypomethylation group. There was significant association between the methylation level at NR4A3 exon 3 CpGi and patient outcome (p=0.034, log-rank test) (Fig. 3C).

### Immunohistochemical staining (IHC)

**Figure 4.**
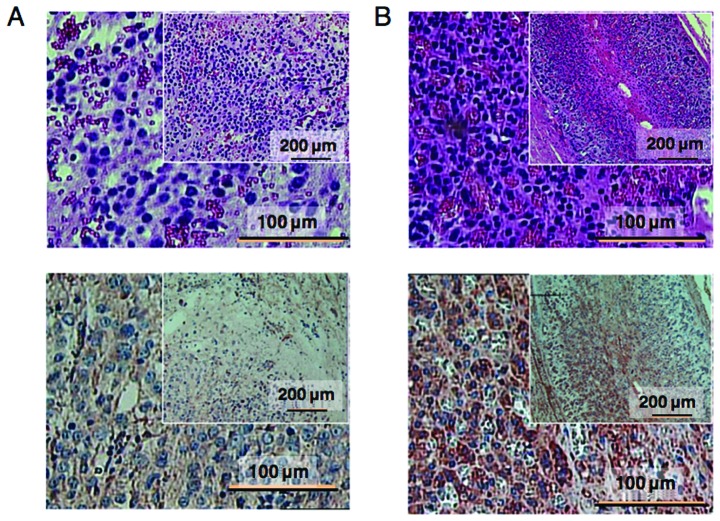
Imunohistochemical analyses of NR4A3 in neuroblastoma and adrenal samples. H&E staining (upper) and immunohistochemical analysis using anti-NR4A3 antibody (lower) were performed for (A) a neuroblastoma section and (B) an adrenal section. Immune reactivity was stronger in adrenal sample than in the neuroblastoma section.

Immunohistochemical analysis using NR4A3 antibody showed a strong signal in adrenal tissue sections and pronounced cytoplasmic staining. On the other hand, faint staining was seen in neuroblastoma specimens (Fig. 4).

### Correlation between NR4A3 exon 3 CpGi methylation and NR4A3 protein expression in neuroblastoma cells

**Figure 5.**
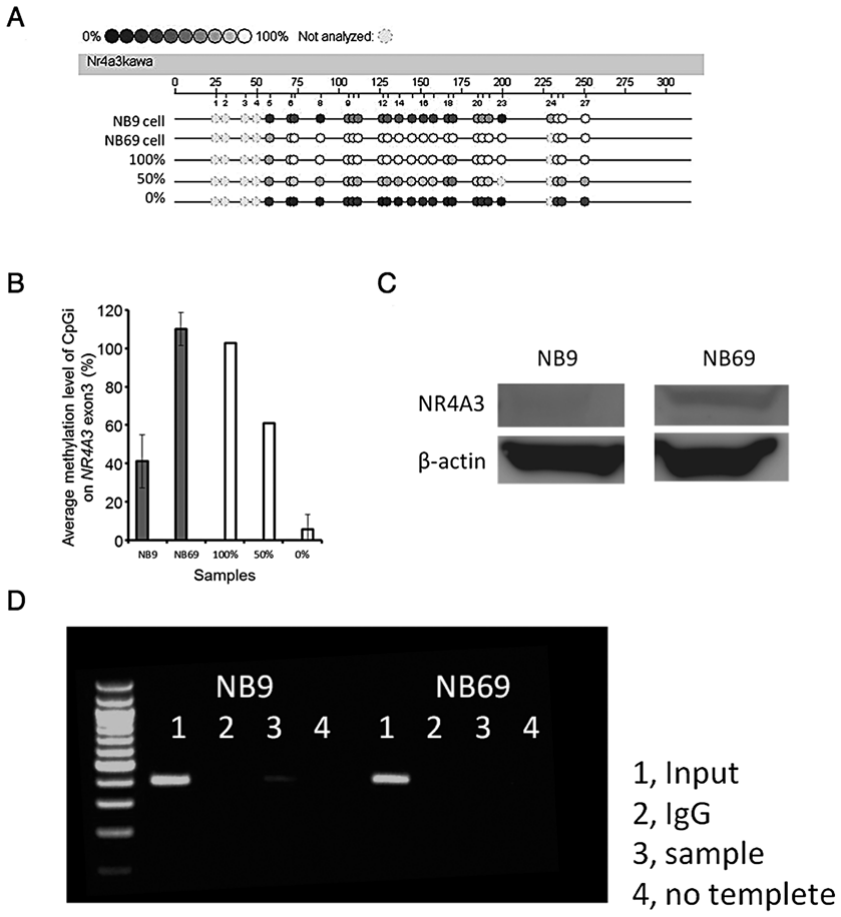
DNA methylation and protein expression levels of NR4A3 in human neuroblastoma cell lines. DNA methylation levels were analyzed quantitatively using Sequenom MassARRAY epiTYPER. (A) Methylation level of each CpG is shown in Epigram. (B) Average methylation level of each CpG site in this region is shown. Gray columns indicate neuroblastoma cell lines and open columns are the standard. Error bars indicate SD. (C) NR4A3 protein expression was analyzed by western blotting. NB69 showed high methylation level of the *NR4A3* gene and expressed NR4A3 protein, while hypomethylated NB9 cells did not express the protein. (D) Chromatin immunoprecipitation assay of NR4A3 exon 3 region. Crosslinked DNA-protein complexes were immunoprecipitated with anti-CTCF antibody and control antibodies, followed by PCR amplification using primers for NR4A3 exon 3. Precipitant from NB9 lysate contained NR4A3 exon 3 region, but no product was amplified in the precipitant from NB69 lysate.

The average methylation level at *NR4A3* exon 3 CpGi in NB9 cells was 44±23.2%, on the other hand, it was 97.1±5.8% in NB69 cell (Fig. 5A and B). Western blot analysis revealed higher expression level of NR4A3 protein in NB69 compared with NB9 (Fig. 5C). This result also indicates that NR4A3 protein expression was correlated with *NR4A3* exon 3 CpGi methylation in human neuroblastoma cell lines.

### Chromatin immunoprecipitation assays

We examined chromatin immunoprecipitation assays to elucidate the mechanism in which methylation level of NR4A3 exon 3 CpGi regulates the expression level. DNA purified from the immunoprecipitated chromatin using anti-CTCF antibody was amplified by PCR for a candidate CTCF binding site of NR4A3 exon 3. In this analysis, the amplified PCR fragment was detected clearly in NB9 cells, but not in NB69 (Fig. 5D).

## Discussion

Many studies have shown that epigenetic alterations, especially aberrant DNA methylation, were involved in the development of various adult tumors (20,21). In neuroblastomas, aberrantly methylated genes, 64% for *THBS1*; 30% for *TIMP-3*; 27% for *MGMT*; 25% for *p73*; 18% for *RB1*; 14% for *DAPK*, *p14ARF*, *p16INK4a* and *CASP8,* respectively, and 0% for *TP53* and *GSTP1* have been reported and the striking differences in methylation status within neuroblastomas has suggested the existence of methylator phenotype, which might be associated with more aggressive forms of neuroblastoma (22,23). Neuroblastoma development is associated with aberration of neural differentiation, and we have reported that aberrant methylation in neuroblastoma at T-/DS-DMR, which plays an important role in differentiation and development (24).

Development of neuroblastoma is related with aberration of the function of neural development factors, such as NGF-dependent tyrosine kinase receptor TrkA activation, relating to differentiation in normal and neoplastic neuronal cells. NR4A are reported to play an important role in the development of neurons (25) and in the regulation of neural function (26). NR4A belongs to a group of early responsive genes, mediating fast response to pleiotropic extracellular stimuli. They bind to NGFI-B response element (NBRE), induce the downstream genes and affect many type of biological function such as oxidative metabolism, cell proliferation, differentiation, apoptosis and dopamine functions in the brain (9,27). In view of their role in brain function, it was reported that NR4A genes, including NR4A3, were induced by psychoactive drugs such as cocaine, morphine, haloperidol and clozapine (28). In cultured cerebellar granule neurons, NR4A transcripts translocated from nucleus to mitochondria during excitotoxicity, contributing to the induction of apoptosis (29).

Our present data about mouse brains also indicate the involvement of NR4A3 and its exon 3 CpGi methylation in the neural development. In the analysis of neuroblastoma specimens, NR4A3 exon 3 CpGi showed low methylation level in neuroblastoma compared with adrenal samples. In addition, hypermethylation of the *NR4A3* exon 3 CpGi was significantly associated with favorable outcome. Since there was a correlation between methylation level at *NR4A3* exon 3 CpGi and *NOR1* expression, our present data suggest that *NOR1* expression level and its genome methylation could be prognostic biomarkers in neuroblastoma.

Methylation of CpG sites at exonic region may be linked to epigenetic remodeling of genomic DNA structure. One of the factors is CCCTC binding factor (CTCF), which is highly conserved in higher eukaryotes. CTCF binds to CTCF-binding sites, and this binding is often regulated by DNA methylation. Of the CTCF-binding sites 45% are located on intergenic, 7% 5′-untranslated region (UTR), 3% exonic, 29% intronic and 20% within 2.5 kb of promoters. H19 DMR is one of CTCF-binding sites and its methylation level is related to epigenetic remodeling, which is co-localized with cohesion. Aberrant regulation of CTCF expression is associated with occurrence of cancers, such as colorectal carcinoma (18,30,31). Our present study showed that CTCF can coimmunoprecipitate with a CTCF binding site within *NR4A3* exon 3 CpGi in NB9 cells but not in NB69 cells. This suggests hypermethylation of *NR4A3* exon 3 CpGi in NB69 cells may inhibit its binding to the CTCF and therefore initiate expression of NOR1. This expression regulation via CTCF binding may be a mechanism of NOR1 down-regulation and involved in advanced neuroblastomas and/or neuronal de-differentiation.

This is the first report indicating that DNA methylation level of *NR4A3* exon 3 CpGi is associated with NOR1 expression and aberrantly methylated in neuroblastomas. In addition, our present data suggest that hypermethylation of this region might be one of the prognostic factors in this tumor. Although further analyses are necessary to elucidate the regulation mechanism of NOR1 expression and its function, our present data strongly suggest that this gene could be involved in neural differentiation and human neuroblastomagenesis.

## Figures and Tables

**Table I.** The tissue samples analyzed.

Patient	Age at diagnosis (month)	INSS	INPC	Shimada classification	Copy nos. of *MYCN*	Prognosis (month)	Methylation level of *NR4A3* exon 3 (%)^a^
Case 1	6	1	NBL, D	FH	1	48S	43.1±5.9
Case 2	10	2	NBL, PD	FH	1	26S	67.1±2.5
Case 3	6	2	NBL, PD	FH	1	48S	20.1±0.9
Case 4	6	4S	NBL, UD	FH	1	48S	43.1±5.9
Case 5	6	2	NBL, PD	FH	1	48S	7.6±0.1
Case 6	7	2	NBL, PD	FH	1	48S	18.4±1.6
Case 7	8	2	NBL, PD	FH	1	48S	83.2±0.4
Case 8	26	4	NBL, PD	UH	10	6R	36.1±1.5
Case 9	36	4	NBL, UD	UH	20	48S	−0.4±8.9
Case 10	30	4	NBL, PD	UH	1	48S	34.3±2.4
Case 11	47	4	NBL, UD	UH	20	4R	8.5±1.5
Case 12	21	3	NBL, UD	UH	150	7R	2.1±7.3
Case 13	98	3	NBL, UD	UH	1	48S	−1.2±7.3
Case 14	77	4	NBL, PD	UH	3	4S	14.7±5.9
Case 15	79	4	NBL, PD	UH	3	35R	39.1±2.2
Case 16	53	4	NBL, UD	UH	4	24R	79.4±0.6
Case 17	20	4	NBL, UD	UH	81	5R	9.1±3.8
Case 18	68	4	NBL, PD	UH	1	10R	5.2±2.5
Case 19	18	4	NBL, PD	UH	119	37S	5.4±4.1
Case 20	33	4	NBL, UD	UH	1	36S	25.6±10.1

INSS, International Neuroblastoma Staging System; INPC, International Neuroblastoma Pathology Committee; NBL, neuroblastoma; PD, poorly differentiated; UD, undifferentiated; FH, favorable histology; UH, unfavorable histology; S, recurrent free survival; R, recurrent.

a Data are shown as mean ± SD.

**Table II.** The primers for quantitative DNA methylation analysis.

Primer name		Sequence
NR3A3-a	Forward	GGAAATTGTTAAGTGTTTTTTTATAT
	Reverse 1	CAACCACCACTTCCTAAAT
	Reverse 2	CGACCACCACTTCCTAAAT
NR3A3-b	Forward	AGTTTTAGAATTTATGTAAGAGGAAAG
	Reverse 1	CACCCAACTATCAAACTC
	Reverse 2	CGCCCAACTATCAAACTC
NR3A3-c	Forward	GAGGTGTTGTTTAGTATTTTTATGTATTTTAAGTAG
	Reverse	CTCACCTTAAAAAAACCCTTACAACC

Each reverse primer has a T7-promotor tag (5′-cagtaatacgactcactatagggagaaggct-3′) for *in vitro* transcription and the forward primer is tagged with a 10 mer tag (5′-aggaagagag-3’) to balance Tm.

## Abbreviations:

T-DMRs: tissue-specific differentially methylated regions;
DS-DMRs: developmental stage differentially methylated regions;
CpGi: CpG island;
E15: 15-day-old embryo;
NB: new born;
AD: 12-week adult;
TrkA: tropomyosin receptor kinase A;
NGF: nerve growth factor;
hMC: homogeneous MassCLEAVE;
MALDI-TOF MS: matrix-assisted laser desorption/ionization time-of-flight mass spectrometry;
Tm: melting temperature;
PCR: polymerase chain reaction;
TBS: Tris-buffered saline;
PBS: phosphate-buffered saline;
CTCF: CCCTC binding factor;
UTR: untranslated region;
AUF1: AU-rich element binding factor 1;
HUR: Hu antigen R
